# Dr. Osborn’s Paper in This Issue

**Published:** 1887-01

**Authors:** 


### DR. OSBORN'S PAPER.

The attention of readers is especially directed to this article as well worthy of a careful perusal, It will furnish food for abundant reflection. We regret that want of space forbids anything like a lengthy comment upon it—hence we leave our readers to draw their own deductions. Dr. Osborn's position in the profession, his long and close observation of clinical phenomena, entitle his opinions on all medical topics to great weight, and for more than a quarter of a century, his contributions to current medical literature have been highly appreciated by the profession; hence in this matter, his observations (meteorological) being so out of the ordinary practice of physicians, and his conclusions so antagonistic to views which, for years, have been accepted as orthodox, we look to see the paper arouse, both an interest in the important subject of the relation of atmospheric pressure to epidemic diseases, and a controversy as to the correctness of his conclusions. The declaration, for instance, that measles and dengue are non-contagious, will strike many with surprise who know what value to put upon the opinion of this Nestor in medicine; while his statement that his observation extending over a period of nearly a half century, convinces him that *diphtheria,* though generally acknowledged by the best authorities to be contagious in Europe, *is not contagious or infectious* in this latitude, will strike many with surprise, and perhaps lead to a new line of thought and action towards this dreaded disease.

